# The utilization of peer feedback during collaborative learning in undergraduate medical education: a systematic review

**DOI:** 10.1186/s12909-019-1755-z

**Published:** 2019-08-23

**Authors:** Sarah Lerchenfeldt, Misa Mi, Marty Eng

**Affiliations:** 1 Department of Foundational Medical Studies, Oakland University William Beaumont School of Medicine, O’Dowd Hall, Room 466, 586 Pioneer Drive, Rochester, MI 48309 USA; 2 Department of Foundational Medical Studies, Oakland University William Beaumont School of Medicine, Kresge Library, #130, 100 Library Drive, Rochester, MI 48309 USA; 3 Department of Pharmacy Practice, Cedarville University, HSC 235, 251 N Main St, Cedarville, OH 45314 USA

## Abstract

**Background:**

Peer evaluation can provide valuable feedback to medical students, and increase student confidence and quality of work. The objective of this systematic review was to examine the utilization, effectiveness, and quality of peer feedback during collaborative learning in medical education.

**Methods:**

The PRISMA statement for reporting in systematic reviews and meta-analysis was used to guide the process of conducting the systematic review. Evaluation of level of evidence (Colthart) and types of outcomes (Kirkpatrick) were used. Two main authors reviewed articles with a third deciding on conflicting results.

**Results:**

The final review included 31 studies. Problem-based learning and team-based learning were the most common collaborative learning settings. Eleven studies reported that students received instruction on how to provide appropriate peer feedback. No studies provided descriptions on whether or not the quality of feedback was evaluated by faculty. Seventeen studies evaluated the effect of peer feedback on professionalism; 12 of those studies evaluated its effectiveness for assessing professionalism and eight evaluated the use of peer feedback for professional behavior development. Ten studies examined the effect of peer feedback on student learning. Six studies examined the role of peer feedback on team dynamics.

**Conclusions:**

This systematic review indicates that peer feedback in a collaborative learning environment may be a reliable assessment for professionalism and may aid in the development of professional behavior. The review suggests implications for further research on the impact of peer feedback, including the effectiveness of providing instruction on how to provide appropriate peer feedback.

## Background

Medical curricula are increasingly integrating collaborative learning [1, 2]. When learning in groups and teams, in which individual students work together to achieve a common goal, such as in team-based learning (TBL) and problem-based learning (PBL), there is an expectation for students to be accountable to both their instructor and peers [3]. One way in which students are held accountable is through the utilization of peer feedback, also known as peer assessment or peer evaluation, which allows students to recognize areas of their strength and weakness as team members. Feedback is essential for learning; it can help students recognize their potential areas of deficiency in their knowledge, skills, or attitude. It is hoped that students use feedback to improve and become effective teammates [4].

There are many advantages to using peer feedback in the medical school curriculum. One advantage is that it can provide a valuable and unique perspective regarding the overall performance of students [5]. Compared to rare encounters with faculty, peers often work together for extended periods of time. Peer assessment is beneficial in assessing areas of proficiency based on multiple observations, rather than one encounter [6]. For this reason, compared to faculty, peers may have the ability to provide more accurate assessments of competencies such as teamwork, communication, and professionalism [7]. Additionally, according to Searby and Ewers, peer assessment may motivate students to produce high-quality work [8]. Overall, peer evaluation may help students improve metacognitive and reflection skills and develop a thorough understanding of coursework by identifying knowledge gaps and reinforcing positive behavior [6, 8–11].

While the literature suggests that peer evaluation can lead to positive outcomes, there are also limitations that must be addressed prior to implementation. Improper or poorly timed peer feedback may impair student relationships and disrupt team function [11]. Poor implementation of peer assessment may create an undesirable class environment that includes distrust, increased competition, or the tendency to exert less effort than they would working alone [3, 12]. Additionally, many students may feel uncomfortable providing peer feedback because of lack of anonymity, potential bias in scores due to interpersonal relationships, and lack of expertise in making assessments [6, 13–15]. Moreover, some students believe peers may be overly nice and not provide honest feedback [13].

Overall, it seems that peer evaluation in a collaborative learning environment has the ability to provide valuable feedback to medical students. It may provide skills necessary to effectively work on inter- and multidisciplinary teams as physicians. It may also increase student confidence and quality of work. The goal of this systematic review was to examine the utilization, effectiveness, and quality of peer feedback in a collaborative learning environment, specifically in undergraduate medical education. The objectives were to determine the role peer feedback plays in student learning and professional development, ascertain the impact peer feedback might have on team dynamics and success, and learn if and how the quality of peer feedback is assessed.

## Methods

### Data sources

A comprehensive literature search was conducted by one of the authors (M.M.). Databases searched included: PubMed, PsycINFO, Embase, Cochrane Library, CINAHL, ERIC, Scopus, and Web of Science. Search terms included index terms (MeSH terms or subject headings) and free text words (see Appendix for complete search strategies for PubMed): ((education, medical, undergraduate [mh] OR students, medical [mh] OR schools, medical [mh] OR “undergraduate medical education” OR “medical student” OR “medical students” OR “medical schools” OR “medical school”) AND (TBL OR “team-based learning” OR “team based learning” OR “collaborative learning” OR “problem-based learning” OR “problem based learning”) AND ((“peer evaluation” OR “peer feedback” OR “peer assessment”) OR ((peer OR peers OR team OR teams) AND (measur* OR assess* OR evaluat*))). The searches were limited to peer-reviewed English-language journal articles published between 1997 and 2017. Overall, the authors felt a 20-year limit was appropriate to ensure the data being evaluated was applicable in undergraduate medical education today.

### Study selection

Duplicates of all articles retrieved were excluded and screened for full-text review if they were original research articles that assessed the use of peer feedback by medical students in a collaborative learning environment during medical school. Editorials, comments, general opinion pieces, letters, survey research studies, and reviews were excluded. All reference lists of selected articles for full-text screening were hand searched for additional relevant articles not discovered in the initial database searches.

Paired reviewers (S.L. and M.E.) screened titles and abstracts of retrieved articles independently. Citations with abstracts that seemed potentially relevant to the selection criteria were included as candidates for full-text screening. The same two reviewers screened these full-text articles independently and in duplicate based on the selection criteria. When a discrepancy arose in article selection between the two reviewers at the full-text article screening stage, the disagreement went to arbitration by a third reviewer (M.M.) who served as a tiebreaker.

### Data extraction and quality assessment

The systematic review was guided by the Preferred Reporting Items for Systematic Reviews and Meta-Analysis Statement (PRISMA) [16] and the Best Evidence Medical Education (BEME) Guide No. 10, “The Effectiveness of Self-Assessment on the Identification of Learner Needs, Learner Activity, and Impact on Clinical Practice” [16, 17]. A standard extraction framework was developed and piloted with a small sample of included studies to abstract and code data from the selected studies. The two authors (S.L. and M.E.) read each article independently and used the extraction framework to extract data. Data was extracted on the country where the study took place, type of course, type of participants, sample size, type of collaborative learning environments (team-based learning, problem-based learning, etc.), and impact and outcomes of peer feedback being evaluated. This information can be found in Table 1. In addition, the types of studies, data sources for peer evaluation, peer evaluation grading criteria, and assessment methods for the quality of peer feedback were also extracted. A qualitative systematic review was conducted due to the heterogeneity of the selected studies in terms of research designs, types of peer feedback, types of student participants, settings, and outcome measures of the impact of peer feedback.

**Table 1:** Student Peer Feedback and Outcomes in the Context of Collaborative Learning Environment

Author (Year)	Country	Type of Course	Participants	Sample Size	Type of Collaborative Learning	Impact/Outcome of Peer Feedback Evaluated	Colthart et al.	Kirkpatrick (from Steinert et al.)
Ambercrombie (2015) [18]	USA	Not well described	Third-year medical students	137	Not described	Significant relationship between medical students’ performance-approach goals and perceptions of grading fairness in Calibrated Peer Review	Grade 2	Level 2
Bryan (2005) [19]	USA	Basic sciences	First-year medical students	213	Dissection groups	Mixed outcomes for student learning and positive outcomes for assessment of and development of professionalism	Grade 2	Levels 1 and 2
Chen (2009) [1]	USA	Basic sciences	First-year medical students	49	Learning teams for course blocks	Inconclusive outcomes for the assessment of professionalism	Grade 3	Level 1
Cottrell (2006) [20]	USA	Basic sciences	First-year medical students	111	PBL	Positive outcomes for the assessment of professionalism	Grade 4	Level 1
Cushing (2011) [21]	UK	Not well described	First-year medical students and nursing students	93	OSCE groups	Positive outcomes for the development of professionalism	Grade 1	Levels 1 and 2
Dannefer (2005) [22]	USA	Not well described	Second-year medical students	97	PBL; small group learning	Positive outcomes for the assessment and development of professionalism	Grade 1	Level 2b
Dannefer (2013) [23]	USA	Not well described	First-year medical students	32	PBL	Positive outcomes for the assessment of professionalism	Grade 2	Level 3
Emke (2015) [24]	USA	Not well described	Second-year medical students	Not described	TBL	Positive outcomes for the assessment of professionalism	Grade 2	Levels 1 and 2a
Emke (2017) [25]	USA	Not well described	Second-year medical students	246	TBL	Positive outcomes for the assessment of professionalism	Grade 2	Level 2
Garner (2010) [26]	UK	Not well described	First-year medical students	30	PBL	Positive outcomes for the assessment of professionalism	Grade 1	Level 2a
Kamp (2013) [27]	Netherlands	Not well described	Second-year medical students	87	PBL	Mixed outcomes for student learning and positive outcomes for team dynamics; impact of the feedback could be increased with individual reflection, goal setting, and face-to-face clarification	Grade 2	Levels 2 and 3
Kamp (2014) [28]	Netherlands	Basic sciences	First-year medical students	242	PBL	Mixed outcomes for student learning	Grade 3	Levels 1 and 2b
Machado (2008) [29]	Brazil	Not well described	First-year medical students	349	PBL	Negative outcomes in student learning (no improvement)	Grade 2	Level 1
Nieder [2005) [30]	USA	Basic sciences	First-year medical students	97	TBL	Inconclusive outcomes for student learning	Grade 2	Level 2b
Nofziger (2010) [31]	USA	Not well described	Second and fourth-year medical students	138	PBL; interviewing groups; lab groups; clinical teams	Positive outcomes for the development of professionalism	Grade 2	Levels 1 and 2a
Parikh (2001) [4]	Canada	Not well described	Fourth-year medical students	103	PBL	Students find peer feedback as a helpful feedback modality; schools would like increase their use of peer feedback	Grade 3	Levels 2a and 3
Parmelee (2009) [32]	USA	Not well described	First-year medical students (also surveyed in their second-year)	180	TBL	Student satisfaction with peer evaluation declined from the first year to the second year	Grade 3	Level 2a
Papinczak (2007*a*) [6]	Australia	Basic sciences	First-year medical students	165	PBL	Positive outcomes for student learning and development of professionalism	Grade 2	Level 1
Papinczak (2007*b*) [33]	Australia	Not well described	First-year medical students	125	PBL	Peer assessment is more accurate than self-assessment; Correlation between peer and tutor feedback scores improved with iterations	Grade 1	Level 2a
Pocock (2010) [34]	UK	Not well described	Second-year medical students	180 (groups)	PBL	Positive outcomes for team dynamics	Grade 1	Level 2a
Reiter (2002) [35]	Canada	Not well described	First-year medical students	36	PBL	Poor correlations between peer assessment compared to assessments from self and tutors	Grade 2	Level 1
Renko (2002) [36]	Finland	Not well described	Fifth-year medical students	49	PBL	Peer observers provided feedback based on analysis of skills used to solve a problem; learned value of careful listening as an outside analyzer	Grade 1	Level 1
Roberts (2017) [37]	Australia	Not well described	First and second-year medical students	633	PBL	Mixed outcomes for the assessment of professionalism; First year students’ ratings provided more reliable than second year students’ ratings	Grade 3	Level 1
Rudy (2001) [38]	USA	Not well described	First-year medical students	97	Interview course groups	Inconclusive outcomes for student learning and positive outcomes for assessment of professionalism	Grade 1	Level 2b
Schönrock-Adema (2007) [39]	Netherlands	Not well described	First-year medical students	278 (1st semester); 272 (2nd semester)	Tutorial groups	Mixed outcomes for the assessment and development of professionalism	Grade 2	Level 3
Sullivan (1999) [5]	USA	Clerkships	Third-year medical students	154	PBL	Compared associations between self, peer, and faculty evaluations and found a moderate correlation between peer and tutor ratings	Grade 1	Level 1
Tayem (2015) [40]	Bahrain	Not well described	Fourth-year medical students	55	PBL	Positive outcomes for student learning, development of professionalism, and team dynamics	Grade 1	Level 1
van Mook (2012) [41]	Netherlands	Not well described	Second-year medical students	307	PBL	Negative outcomes for team dynamics and the assessment of professionalism	Grade 2	Level 3
Vasan (2009) [42]	USA	Basic sciences	First-year medical students	355	TBL	Positive outcomes for student learning and negative outcomes for team dynamics	Grade 1	Level 1
White (2012) [43]	Canada	Clerkships	Third-year medical students	116	Clinical teams	Peers provided more feedback on the “team member” (i.e. work ethic, communication, leadership) and “person” (i.e. compassion, respect, humor) domains of clinical performance compared to other assessors	Grade 1	Level 2a
Zgheib (2016) [44]	Lebanon	Not well described	First and second-year medical students	102	TBL	Positive outcomes for student learning, team dynamics and development of professionalism	Grade 2	Levels 2 and 3

Out of 31 studies, 11 provided students with instruction on peer feedback. None of the included studies provided any information regarding if faculty evaluated the quality of peer feedback. Eight studies used peer feedback as formative (ungraded) assessment, 4 studies for summative (graded) assessment, 2 for both formative and summative assessments, and the rest of included failed to describe how peer feedback was used for outcome assessment

We used an adapted Kirkpatrick evaluation model [45] to classify the effectiveness/impact of peer feedback in this review. There are six levels ranging from level 1 (Reaction - participants’ views on the learning experience) to level 4B (Results – improvement in student learning as a direct result of their educational intervention) [45].

We used a grading system, the Gradings of Strength of Findings of the Paper by Colthart et al. to score the strength of study findings on a scale of 1 to 5 [17]. Articles scored 4 or above on the strength of findings were considered to be higher quality papers. Articles scored 3 had conclusions that could probably be based on the results. Articles graded as 2 or 1 were regarded as, “results ambiguous, but there appear to be a trend,” or "No clear conclusions can be drawn (not significant). Two authors (S.L. and M.E.) independently carried out data extraction and quality appraisal. Three authors (S.L., M.E., and M.M.) reviewed and discussed discrepancies and came to a consensus.

## Results

### Retrieved studies

**Fig. 1:**
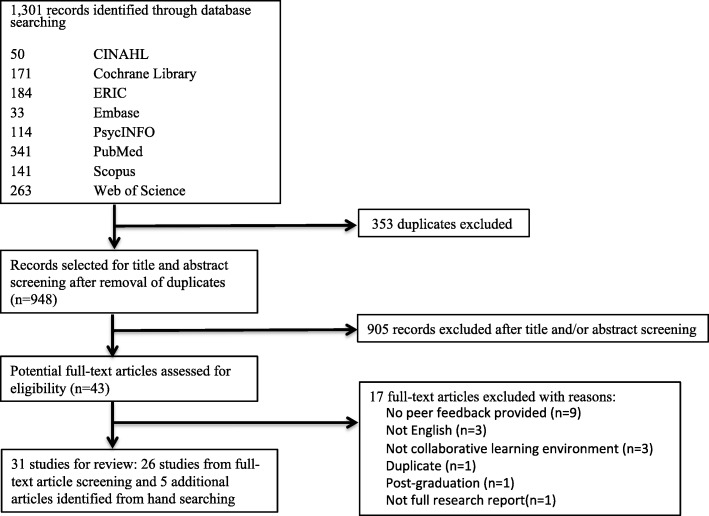
Flowchart of Study Selection Process

A total of 1301 articles were returned from the literature search. After removal of duplicates, 948 remained. A further 905 articles were excluded after title and/or abstract screening leaving 43 for full-text review. Of these, 26 articles were included in this review in addition to 5 further articles identified through hand searching of references (31 in total). Further details shown in PRISMA flowchart (Fig. 1).

### Study characteristics

Of the included studies, the majority were completed in the United States (*n* = 14), followed by the Netherlands (*n* = 4), and Australia (*n* = 4), Canada (*n* = 3), and the United Kingdom (*n* = 3). Other countries included Bahrain, Brazil, Finland, and Lebanon.

The sample size for the studies ranged from 30 to 633 students. Most studies included first-year (*n* = 18) and second-year (*n* = 9) medical students. Peer feedback was evaluated through collaborative learning activities integrated into preclinical courses (*n* = 7) and clerkships (*n* = 2); although many studies did not provide clear information on where peer feedback was evaluated in the curriculum. PBL (*n* = 18) and TBL (*n* = 6) were used in the collaborative learning setting.

The research methodology of selected studies included 15 quantitative, 3 qualitative, and 13 mixed methods (defined as including both quantitative and qualitative data). Most studies utilized quantitative questionnaires to obtain data (*n* = 26). Other data sources included narrative comments (*n* = 14), focus groups (*n* = 5), open-discussions (*n* = 1) and individual interviews (*n* = 1). Many studies did not describe the grading criteria for the peer feedback (*n* = 17); of those that did, most were formative (ungraded) in nature (*n* = 8), while some courses included formative and summative (graded) peer feedback (*n* = 2) and some only included summative peer feedback (*n* = 4). Eleven studies reported that students received instruction on how to provide appropriate peer feedback, but no studies provided descriptions on whether or not the quality of feedback was evaluated by faculty.

A total of 17 studies evaluated the effect of peer feedback on professionalism in some manner. There were 12 studies that evaluated the effectiveness of peer feedback for the assessment of professionalism. Of those 12 studies, eight had positive results, two had mixed results, one had negative outcomes, and one was inconclusive. Eight studies evaluated the use of peer feedback for the development of professional behavior, in which seven had positive results and one had mixed results. Ten studies examined the effect of peer feedback on student learning, in which four had positive results, three had mixed results, one had negative outcomes, and three were inconclusive. In addition, there were six studies that examined the role of peer feedback on team dynamics. Of those studies, four had positive outcomes, whereas two had negative results. Table 1 contains more details about the selected articles, including sample size, participants, and setting.

## Discussion

This systematic review examined the role of peer feedback in a collaborative learning environment in undergraduate medical education. It revealed a large number of variations in research design, approaches to peer feedback, and definitions of outcome measures. Due to the heterogeneity of these studies, it was difficult to assess the overall effectiveness and utility of peer feedback in collaborative learning for medical students. Despite these differences, the overall outcomes for most of the studies were positive.

### Assessment of professionalism

The professional development of medical students is an essential aim of the medical school curriculum [20]. Peer feedback may provide reliable and valid assessment of professionalism. In the systematic review, several studies reported positive outcomes for the assessment of several aspects of professionalism. Chen and colleagues reported peers were able to accurately evaluate respect, communication and assertiveness of student leaders [1]. Dannefer and colleagues found that peer feedback was consistently specific and related to the professionalism behaviors identified by faculty as fundamental to practice. In addition, they found that peers often gave advice on how to improve performance, which is often considered a type of feedback that can result in positive changes in behavior [23, 46]. Emke and colleagues found that the use of peer evaluation in TBL may help identify students who may be at risk for professionalism concerns [25].

Although many studies had encouraging results in regards to the use of peer feedback for the assessment of professionalism, some studies did not show positive results. For example, Roberts and colleagues found that peer assessment of professional learning behavior was highly reliable for within-group comparisons, but poor for across-group comparisons, stating that peer assessment of professional learning behaviors may be unreliable for decision making outside a PBL group [37].

### Development of professionalism

According to Emke and colleagues, professional behavior is a cornerstone of the physician-patient relationship, as well as the relationship between colleagues working together on a multidisciplinary team [25]. Many studies reported positive outcomes when assessing the development of professionalism. Nofziger and colleagues found that 65% of students reported important transformations in awareness, attitudes, and behaviors due to high quality peer assessment [31]. Papinczak and colleagues found that peer assessment strengthened the sense of responsibility that group members had for each other, in which several students were enthusiastic and committed to providing helpful and valid feedback to support the learning of their peers [33]. In addition, studies by Tayem and colleagues and Zgheib and colleagues reported improvements in communication skills, professionalism, and ability to work on a team [40, 44].

### Student learning

Peer assessment may also be beneficial for student learning. Tayem and colleagues reported that a large percentage of students that participated in peer assessment in TBL felt that peer assessment helped increase their analytical skills as well as their ability to achieve their learning objectives and fulfill tasks related to the analysis of problems [40]. Zgheib and colleagues noted that students learned how to provide peer evaluations that were specific and descriptive, and expressed in terms that were relevant to the recipient’s needs, preparing them for their role in providing feedback as future physicians [44]. While peer assessment may be an important tool to improve student learning, some outcomes were mixed. For example, Bryan and colleagues stated that students were capable of commenting on professional values, but may lack the insight to make accurate evaluations. They recommended that peer-assessment should be used as a training tool to help students learn how to provide appropriate feedback to others [19].

### Team dynamics

Students must learn how to effectively work on a team to become successful physicians [47]. Peer feedback may be valuable in the development of medical students in becoming effective team members. For example, Tayem and colleagues reported that a large proportion of participants agreed or strongly agreed that their respect towards the other group members and desire to share information with them had improved. The students also agreed that they had become more dependable as a result of peer assessment [40].

### Grading criteria

In many cases, the grading criteria were not clearly described. Of the studies that described the grading criteria, the majority felt that peer feedback was appropriate to use in a formative, or ungraded, manner. Although most studies did not describe the details of their grading criteria, the literature supports the use of formative and summative assessments for peer feedback evaluations. According to Cestone and colleagues, peer assessment in TBL can provide formative information to help individual students improve team performance over time. Formative peer feedback can also aid in the development of interpersonal and team skills that are very important for success in future endeavors [3]. According to Cottrell and colleagues, one evaluation is not adequate, in which implementing the peer assessment multiple times and across a variety of learning contexts lends students opportunities to make formative changes for improvement [20].

### Instruction on how to provide high-quality peer feedback

Providing effective feedback for peers is a skill that should be developed early in medical and health professions education [1]. According to Burgess and colleagues, peer review is a common requirement among medical staff, but since formal training in providing quality feedback is not common in the medical school curriculum, physicians are often not well prepared for this task [48].

Out of 31 studies, 11 described the instruction that was provided to students on how to provide effective peer feedback. These studies stressed the important of training students how to provide feedback for strengths as well as areas needing improvement. Without any guidance or training on how to provide peer feedback, students may feel confused or not know how to assess peer properly. In the study by Garner and colleagues, they found that students were unclear about the purpose of peer assessment and felt the exercise was imposed upon them with little preparation or training, creating anxiety for individual students [26]. Nofziger stated that students should receive training to provide specific, constructive feedback and that the institutional culture should emphasize safety around feedback, while committing to rewarding excellence and addressing concerning behaviors [31]. Better instruction on how to provide appropriate feedback may make the goals of peer feedback clearer as well as decrease student anxiety when performing assessments of their peers.

### Evaluation of feedback quality

This systematic review also evaluated whether or not the quality of peer feedback was assessed by students or faculty. Most included studies failed to address the importance of the evaluation of peer feedback by students, including the potential importance of educators reviewing the feedback quality. Faculty should be trained on how to evaluate the quality of feedback to make sure students are effectively assessing their peers, as well as help remediate students who are not performing to the best of their abilities.

### Limitations

Our systematic review was limited to published studies in English. As a result, potential publication bias and language bias may have been introduced to the review. Other reporting biases in these selected studies may contribute to biased conclusion of the study reports. These biases could potentially present a threat to the validity of any type of review including this systematic review. The presence of negative and inconclusive studies and small effects do not support publication bias. A full analysis for publication bias is not feasible and thus, cannot be ruled out. An advantage is that this review is one of the first to apply standard methods of evaluating both study outcomes and quality of the existing literature related to peer feedback during collaborative learning in undergraduate medical education. The descriptive methods inherently limit potential conclusions that may be drawn from reported results. These proved to be challenging to apply because the majority of the studies were descriptive in nature.

With regards to strength of methods, an overwhelming majority of studies received lower ratings. This pattern was often due to outcomes not specified a priori, no power discussion, unclear primary study objective, or discordant conclusion with originally stated study aim. A few studies seemed to use validated surveys to measure student perceptions but then reported outcomes measures without interpretation of results.

The strongest studies were conducted by Chen, Cottrell, Kamp, Parikh, and Roberts [1, 4, 20, 28, 37]. The strength of Cottrell 2006 was the report of internal consistency of the rubric and generalizability of the results [20]. Kamp 2014 had a sound pre-post design. However, they did not report a power calculation [28].

Finally, the most common study outcomes were evaluated at levels 1 and 2 according to the modified Kirkpatrick Evaluation Model [45]. These corresponded mostly to the feasibility of doing peer feedback in a variety of learning contexts. The second most common outcome was student perceptions of peer feedback. Overall, students had favorable perceptions. Concordance between self and others’ feedback was considered level 1. Lastly, professionalism was the most common level 3 outcome reported.

## Conclusions

The objectives of this systematic review were to determine the role peer feedback plays in student learning and professional development, ascertain the impact peer feedback might have on team dynamics and success, and learn if and how the quality of peer feedback is assessed. Our review highlights the heterogeneity of the current literature regarding the use of peer feedback in undergraduate medical education. Overall, peer feedback in a collaborative learning environment may be a reliable assessment for professionalism and aid in the development of professional behavior. Many studies felt that peer feedback was appropriate to use in a formative manner. Most studies do not address the importance of the quality of peer feedback provided by students. Due to the wide variations in the outcomes defined by these studies, it may be beneficial to have more standardized definitions for student learning, team-dynamics, and professionalism. Despite the large variety of contexts and outcomes studied, there seems to be a consistent message. Peer feedback in collaborative learning is feasible and may be useful. Steps to ensure success include training faculty and students on peer feedback methods and purpose. Because developing and implementing peer feedback systems takes significant energy and resources, further studies should increase in methodologic reporting rigor and seek to expand outcomes to include, but not be limited to, quality of peer feedback (including the effectiveness of providing faculty and student training), the effect on academic performance, institutional culture, and benefits to future employers and patients.

## Data Availability

Not applicable

## Appendix

### Search Strategies for PubMed Search

(education, medical, undergraduate [mh] OR students, medical [mh] OR schools, medical [mh] OR “undergraduate medical education” OR “medical students” OR “medical student” OR “medical schools” OR “medical school”) AND ((cooperative behavior[mh] AND educational measurement [mh] AND group processes [mh] AND learning [mh]) OR (“cooperative behavior” AND “educational measurement” AND “group processes” AND learning) OR “peer evaluation” OR “peer feedback” OR “peer assessment” OR ((peer OR peers OR team OR teams) AND (measur* OR assess* OR evaluat*))) AND (TBL OR “team-based learning” OR “team based learning” OR “collaborative learning” OR “problem-based learning” OR “problem based learning”)

## Abbreviations

M.E.: Mary Eng (author)
M.M.: Misa Mi (author)
PBL: Problem-based learning
PRISMA: Preferred Reporting Items for Systematic Reviews and Meta-Analysis Statement
S.L.: Sarah Lerchenfeldt (author)
TBL: Team-based learning
